# What Genetics Has Told Us and How It Can Inform Future Experiments for Spinal Muscular Atrophy, a Perspective

**DOI:** 10.3390/ijms22168494

**Published:** 2021-08-06

**Authors:** Anton J. Blatnik, Vicki L. McGovern, Arthur H. M. Burghes

**Affiliations:** Department of Biological Chemistry & Pharmacology, The Ohio State University Wexner Medical Center, Rightmire Hall, Room 168, 1060 Carmack Road, Columbus, OH 43210, USA; blatnik.12@buckeyemail.osu.edu (A.J.B.III); mcgovern.43@osu.edu (V.L.M.)

**Keywords:** SMA, spinal muscular atrophy, genetics, biochemistry, SMN function, SMN missense mutants, survival motor neuron, suppressor screen, motor neuron

## Abstract

Proximal spinal muscular atrophy (SMA) is an autosomal recessive neurodegenerative disorder characterized by motor neuron loss and subsequent atrophy of skeletal muscle. SMA is caused by deficiency of the essential *survival motor neuron* (SMN) protein, canonically responsible for the assembly of the spliceosomal small nuclear ribonucleoproteins (snRNPs). Therapeutics aimed at increasing SMN protein levels are efficacious in treating SMA. However, it remains unknown how deficiency of SMN results in motor neuron loss, resulting in many reported cellular functions of SMN and pathways affected in SMA. Herein is a perspective detailing what genetics and biochemistry have told us about SMA and SMN, from identifying the SMA determinant region of the genome, to the development of therapeutics. Furthermore, we will discuss how genetics and biochemistry have been used to understand SMN function and how we can determine which of these are critical to SMA moving forward.

## 1. Introduction

Proximal spinal muscular atrophy (SMA) is an autosomal recessive neurodegenerative disorder that affects 1 in 10,000 individuals [1,2,3]. The disorder is caused by *survival motor neuron* (SMN) protein deficiency, resulting from loss of function mutation or deletion of the *SMN1* gene, but retention of the companion *SMN2* [4]. Until recently, SMA was the most common genetic cause of infant death [5], but in the last several years, three therapies have proven remarkably successful in challenging this claim [6,7,8]. Despite the efficacy of these therapeutics, we still do not know why deficiency of SMN results in SMA. Genetic and biochemical experiments have determined that SMN deficiency causes SMA. This perspective will detail how genetics can be used in the future to study the function of SMN and validate downstream targets that are altered by SMN deficiency.

## 2. Identifying *SMN1* and *SMN2* as the SMA Determinant Genes

**Figure 1:**
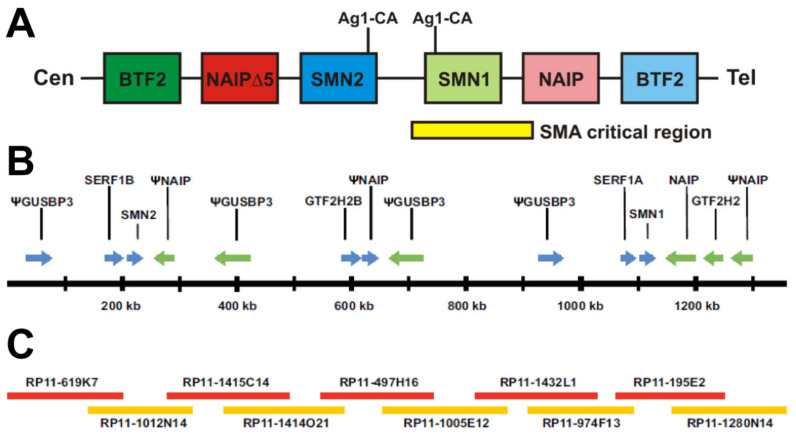
Genomic organization of *SMN1* and *SMN2*. (**A**) Original construction of the SMN region showing inverted duplication. The centromeric end of the chromosome is given on the left and telomeric end on the right. Ag1-CA markers are indicated above with lines to their approximate locations in *SMN1* and *SMN2*, thus suggesting inverted duplication. Further genes in the region, BTF2 and NAIP, are also given, further suggesting inverted duplication. (**B**,**C**) Map of the SMA region that was assembled using overlapping clones that originate from the same chromosome. (**B**) Map of the region showing all genes and their orientation. *SMN1* and *SMN2* are in the same orientation and are approximately 848 kb away from each other. Lying between *SMN1* and *SMN2* are 2 *NAIP* pseudogenes (one containing *NAIP* exons 6–17 and the other contained *NAIP* exons 3 and 6–9) and 2 *GUSBP3* pseudogenes, as well as *SERF1A* and *GTF2H2B*. Pseudogenes are indicated with a (Ψ). (**C**) Overlapping clones used to construct the region. The smallest overlap between clones was 32,833 bp while the average overlap was 71,850 bp. There was only a single mismatched base pair out of all the overlapping regions.

SMA is an autosomal recessive neurodegenerative disorder characterized by the loss of spinal motor neurons and subsequent atrophy of muscle [9]. The disorder can be divided into five types, ranging in severity from Type 0, the most severe showing onset at birth, to Type 4, the mildest with onset in adulthood [9,10]. Early studies of patient pedigrees indicated different SMA types could occur in the same family, suggesting mutations in the same locus could give rise to all types of SMA [11,12,13,14,15,16]. The first steps in the molecular era were to map the causative gene using linkage analysis, which started by identification of genetic markers that segregated with the SMA phenotype [17,18,19,20]. The region was then narrowed with flanking markers until those markers were very close to the gene with no recombinants. It was interesting to find that the markers with no recombinants were in a region that was, at the very least, duplicated, indicating there were multiple copies of the gene of interest within that region. It later became clear this was due to duplication and insertion [4]. In 1995 it was determined that patients of differing SMA types all had mutations in a single gene, *survival motor neuron 1* (*SMN1*) [4]. However, a nearly identical gene, *SMN2*, was always retained [4]. The SMA region is complex, and the multiple possible structures were not well represented in the genome assembly [4,21,22]. *SMN1* and *SMN2* were originally reported to exist in an inverted duplication; however, in our recent revisit of the region, we found a tandem duplication with an additional insertion (Figure 1) [4,22]. Our approach ensured assembly from one chromosome by requiring all single nucleotide polymorphisms (SNPs) aligned in a relatively large overlap.

Both *SMN1* and *SMN2* were predicted to make SMN protein, however, SMN cDNAs derived from SMA patients showed the predominant transcript from *SMN2* was truncated and lacked exon 7 (SMN∆7) [4,23,24]. A single nucleotide change from C to T was identified within *SMN2* exon 7 affected the inclusion of exon 7 during pre-mRNA splicing [25,26]. This truncated SMN∆7 protein had decreased ability to oligomerize, resulting in degradation of the unstable product [27,28]. This was confirmed in SMA patient lymphoblasts where an SMN∆7 specific antibody could not detect the truncated protein [29]. Thus, in the case of SMA, where patients have a loss-of-function mutation or deletion in *SMN1* but retention of *SMN2*, patients are reliant on the small amount of full-length SMN produced from *SMN2* resulting in SMN protein deficiency [23,30,31]. The variable presentation of SMA in the clinic is then largely explained by duplications and additions of *SMN2* copies. As such, more copies of *SMN2* result in increased full-length SMN protein and a less severe phenotype [32]. In fact, our model of the SMA region as a straightforward duplication with additional insertion is compatible with the prediction that an unequal cross over results in loss of *SMN1* with duplication of *SMN2*, thus explaining a mechanism for increased *SMN2* copies in SMA patients [22]. The genetic understanding of *SMN2* as a modifier of SMA, combined with the biochemical understanding that more copies of *SMN2* results in increased full-length SMN protein, indicated a strong target for therapeutics to increase full-length SMN protein.

## 3. Genetics Informed SMA Therapeutics

As SMA is caused by SMN protein deficiency, the most obvious therapeutic approach was to increase SMN levels [30,31]. Since SMN deficiency is caused by inefficient inclusion of exon 7 in *SMN2* transcripts, and that *SMN2* is present in the entire patient population, there was strong rationale to screen for compounds that modulated *SMN2* splicing. Indeed, it soon became clear that the C to T change in exon 7 removed an exon inclusion enhancer. In addition, several elements in both introns and exons that either enhanced exon 7 inclusion or inhibited exon 7 inclusion were identified including the intron splice silencer ISS-N1, which is the target of the antisense oligonucleotides (ASO) therapeutics [33,34,35,36]. Spinraza (Nusinersen) and Risdiplam (Evrysdi) were discovered and developed through screens for increasing full-length SMN production from *SMN2* [34,37,38,39,40,41]. Both Spinraza and Risdiplam target intron 7; however, they enhance exon 7 inclusion by two different mechanisms. Spinraza is an ASO that targets ISS-N1 blocking inhibition of spliceosome recognition by hnRNPA1. Risdiplam is a small molecule drug that binds and stabilizes an unpaired adenine in the exon 7 5′ splice site which allosterically promotes binding of the U1-C zinc finger and U1-snRNP [42,43,44]. This acts by turning the weak 5′ splice site into a stronger one. Both therapies increase full-length SMN protein levels, survival, and motor function in SMA mice when delivered presymptomatically [38,39,40,44,45,46]. Clinical trials show these drugs are safe and efficacious [6,8,47,48]. In fact, presymptomatic two and three copy *SMN2* SMA patients who received Spinraza achieve major motor milestones like sitting independently and walking with or without assistance [48]. Additionally, for those patients who receive treatment after onset of overt symptoms, both drugs have been shown to delay the progression of SMA [8,47]. The drugs are currently approved for treatment of SMA patients by both American and European medical authorities.

The presence of *SMN2* in every SMA patient gives a unique target and an advantage in the development of gene therapy as well. The classic use of gene therapy delivers a cassette to a particular tissue(s) and expresses a gene product in *trans* to the genome. In SMA, this is performed by expression of the SMN cDNA from the ubiquitous chicken beta-actin (CBA) promoter, delivered by packaging it in adeno-associated virus serotype 9 (AAV9) [49,50,51]. The major advantage for a gene therapy approach in SMA is that patients already produce some SMN, thus reducing the possibility of immunological reaction to the introduced protein. AAV9-SMN gene therapy also increases survival and motor function in SMA mice when administered presymptomatically [50,52]. It is approved for treatment of SMA in the United States under the label Zolgensma (Onasemnogene abeparvovec). A single intravenous dose of Zolgensma resulted in similar achievement of major motor milestones consistent with Spinraza and Risdiplam [7]. Additionally, clinical trials with Zolgensma also indicate presymptomatic treatment yields the best results [7,53,54]. Trials are currently investigating an intrathecal delivery method [55].

Furthermore, *SMN2* can be manipulated to act like *SMN1* in what could potentially become a permanent fix to the level of SMN. There are many approaches that can be considered for manipulating *SMN2*, the most obvious of which is using an adenine base editor (ABEs) which can convert the T change back to a C, thus making *SMN2* into a *SMN1* gene [56,57,58]. Another possibility is the modification of negative regulators of *SMN2* exon 7 inclusion such as ISS-N1, which when disrupted effectively turns *SMN2* into *SMN1*, or at least *SMN1*-like [59]. This is an exciting prospect as it moves from a conventional gene therapy to the use of Cas or modifying base editors to make *SMN2* function like *SMN1* permanently. This will give the correct expression levels and not be in danger of loss of the expression cassette from the target tissue, or shutdown of expression by epigenetic mechanisms. The disadvantages for these approaches are the expression of the foreign enzymes, the off-target effects involved in the guide RNA-targeting mechanisms, in some cases the efficiency of the process, and the size constraints for packaging the cassettes in AAV. The future could entail a new breed of therapies that can truly fix the base defect. It is hoped that these could also be less expensive as technology moves forward and manufacturing of AAV gene therapy vectors advances.

## 4. How Do SMN Mutations Work

**Figure 2:**
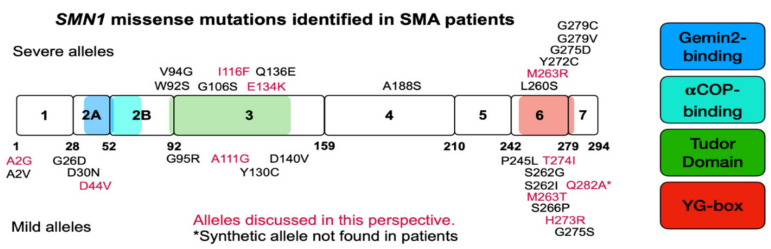
Pictorial representation of SMN exons, domains, and mutations identified in SMA patients. SMN coding region depicted in block form with number designation for exons. Numbers underneath blocks designate the amino acids that end each exon, or are before the translation termination signal. Severe SMN missense mutations are given above blocks and mild mutations are given below blocks, in the approximate location they occur. The mutant alleles given in magenta are discussed within this manuscript. The colored boxes indicate domains of SMN in order from left to right: Gemin2-binding, alpha-COP-binding, Tudor Domain, and YG-box oligomerization domain. Please consult Table 1 for more detailed information about each missense mutation, the SMA type, *SMN2* copy number, and publication.

The vast majority of SMA cases result from a large deletion in *SMN1*; thus patients are entirely reliant on the full-length SMN produced from *SMN2* [32,60]. However, 1% of SMA cases are caused by a loss of function missense mutation in *SMN1* [32,60]. In every instance, missense mutations in *SMN1* are always found in the presence of the *SMN2* gene. Interestingly, some of these mutations are found in patients who present with milder symptoms than is expected from their *SMN2* copy number; thus we refer to those variants as mild SMN missense mutations [61]. Conversely, there are missense mutations in *SMN1* that do not modify the expected SMA phenotype as suggested by the *SMN2* copy number [61] and these are referred to as severe mutations. Severe SMN missense mutations are predominantly found in the SMN Tudor domain—important for Sm-ring assembly (exon 3)—and the oligomerization domain (exon 6), both highly conserved regions of the SMN protein [61]. Thus, some severe mutations interfere with dimerization and disrupt SMN function. Missense mutations have been found in every SMN exon except exon 2B and exon 5 (Table 1). A pictorial representation of Table 1 is given in Figure 2.

As mild SMN missense mutations can alter SMN severity, it was suggested these alleles are partially functional. We addressed this question by first expressing an SMN missense allele in a cell line conditionally deleted for endogenous full-length Smn [77]. We generated an immortalized mouse embryonic fibroblast (iMEF) line that contains the *Smn* knockout exon 2A neomycin insertion allele (*Smn^−^*) and the floxed Smn exon 7 allele (*Smn^F7^*) [77,78,79]. When Cre-recombinase is expressed in these lines, *Smn* exon 7 is deleted (*Smn^D7^*), resulting in loss of functional Smn production in the cell and cell death [77,79]. Firstly, this indicates Smn is essential for cell survival and secondly, that *Smn^D7^* does not make an Smn capable of essential function. We cloned the mild SMN missense mutant cDNAs A2G, D44V, A111G, T274I, and the severe SMNE134K missense mutant into mammalian expression constructs, transfected these into our *Smn^−/F7^* iMEFs, and selected stable expressing lines [77]. When treated with Cre, removing the endogenous source of full-length wild-type Smn, these cells also die [77]. We found that none of the missense mutations on their own could rescue the loss of Smn protein and the cells died.

Next, we expressed mild SMN missense alleles transgenically to determine if overexpression could rescue embryonic lethality in *Smn^−/−^* mice [80,81,82,83]. Mice expressing the mild SMN missense mutants A2G, D44V, A111G, T274I, Q282A, or the severe SMNI116F missense mutant on an *Smn^−^* background were not viable [80,81,82]. These experiments indicated that missense mutations in *SMN1* are incapable of the essential function of SMN in the absence of full-length SMN protein. It should be noted that missense mutations in *C. elegans* and *Drosophila* do not behave in the same manner as those in mice and humans [64,67,82,84,85,86]. For instance, the orthologous mutants for SMND44V can rescue the *Smn* null animals in worms and flies [85,86]. Additionally, expression of the orthologous severe SMNI116F mutant rescues *Smn* null in *Drosophila* [86]. We postulate that this incongruence between orthologous SMN missense mutant activity in these models may be due to a difference in the downstream targets of SMN function. Thus, it is important to test targets found in *C. elegans* and Drosophila in mammalian systems to ensure translatability.

After establishing that missense mutations in *SMN1* are nonfunctional in mammals in the absence of full-length SMN, we asked, how are these mutations able to modify the SMA phenotype in patients? The functional mechanism likely involves oligomerization. SMN is an oligomeric protein and several experiments have indicated oligomerization is necessary for SMN function [27,28,77,82,83]. Furthermore, the predominant product of *SMN2*, the truncated SMN∆7 protein, inefficiently oligomerizes and is rapidly degraded [27,28]. Finally, to date all missense mutations that prevent SMN oligomerization are severe and all mild SMN missense mutants can oligomerize [27,87,88].

Previously, other oligomeric proteins have been shown to perform intragenic complementation, a situation in which two mutant proteins of a single gene form a heteromeric complex that has increased function when compared to a complex composed of each mutant alone [89]. This is evident with loss of function mutations in argininosuccinate lyase and calpain-3 [89,90,91,92]. We have demonstrated that intragenic complementation occurs with the SMN protein. Expression of all mild SMN mutants A2G, D44V, A111G, T274I, or Q282A in the presence of full-length SMN protein produced by *SMN2* rescued weight, survival, and electrophysiology in *Smn* null mice [80,81,82]. This implies the mechanism for modification of the SMA phenotype in patients who have a mild SMN missense mutation lies in the ability of the missense mutant protein to form a functional SMN oligomer with the small amount of SMN produced from *SMN2*. This data strongly suggests the functional unit of SMN in the cell is the oligomer and not the monomer. Furthermore, we have confirmed in our iMEF cell system that expression of two SMN missense mutants also rescues cell survival in the absence of full-length wild-type SMN [77,83]. Notably, in iMEFs the expression level of SMN protein in some of the dually expressing SMN missense mutant iMEFs is less than has been tested for each SMN missense mutant expressed singly [77]. This means that rescue of iMEF survival is due to the presence of the SMN missense mutants and not merely due to a nominal increase in SMN protein abundance.

Recently a structure of the SMN oligomer has been determined in which SMN first dimerizes through the glycine zipper interface and then further oligomerizes in an antiparallel stacking formation to form tetrameric and octameric SMN oligomers [88]. Interestingly, SMN readily exists in tetrameric oligomers in *pombe*; however, gel filtration indicates human SMN adopts an octameric complex composed of a dimer of tetramers [88,93,94]. The authors clearly show severe SMN missense mutations that disrupt initial dimerization through the glycine zipper interface, abrogate higher order SMN oligomerization. The authors also show that mild SMN missense mutations, M263T and T264I that do not prevent SMN dimerization, disrupt the antiparallel stacking interaction, and shift SMN oligomer speciation away from the octameric and tetrameric forms and towards the dimer. This is interesting because the SMNM263T mutant which prefers the dimeric state was found in a patient who presented with Type 2 SMA, while having only one copy of *SMN2* [73,88]. Furthermore, SMNT274I was found in a patient who presented with milder Type 3 SMA, while having only one copy of *SMN2*, and the SMNT274I oligomers preferentially adopt the dimeric and tetrameric states [72]. This indicates that first, the SMNM263T and SMNT274I mutants can interact with wild-type SMN produced from *SMN2* and second, higher order oligomeric formation has an impact on the functional output of SMN. Our own experiments show that expression of the SMNT274I mutant does not rescue lethality of a *Smn* null when expressed in cells or mice, but rescue does occur when SMN from *SMN2* or SMNA111G is present [77,82,83]. Thus, a homomeric mutant does not allow the formation of higher order oligomers but the addition of a wild-type domain does. Interestingly, the association of Gemin8 with SMNM263T and SMNT274I homomers is reduced, suggesting antiparallel stacking and higher order oligomer formation is important for proper SMN complex integrity and function [88]. It should be noted that while SMNT274I fully rescues SMA mice when *SMN2* is present, this is not the case in humans as SMNT274I or SMNM263T, with one copy of *SMN2*, does result in SMA in humans. Thus, these oligomers have reduced function compared to wild-type SMN complexes in humans [72,82].

The combination of genetics and biochemistry has given insight into SMN and its function, but many questions remain regarding the SMN protein, its structure, function, and domains. For instance, we do not know the true stoichiometry of the SMN complex, nor have we determined how SMN and the Gemins are spatially oriented within the complex. Additionally, we determined the SMNA2G mutant does not complement the SMNT274I mutation whereas both SMND44V and SMNA111G do complement [77]. This suggests an interaction or shared function between the extreme N-terminus and C-terminus of SMN. Could the N- and C-terminus share a common interaction with Gemin8 or Gemin3 and stabilize the association of the fully assembled complex as a whole? We propose that a combination of genetics and biochemistry can continue to find answers to these questions.

## 5. Suppressor Screens Which Have Been Informative in Other Neurodegenerative Disorders

Here, we discuss two suppressors screens that have been informative in determining the critical function or targets for therapy in other neurodegenerative diseases. We focus on Spinocerebellar ataxia type 1 (SCA1) [95], where suppressors inform on the mechanism of the disease [96,97,98,99], and Rett Syndrome caused by MECP2 deficiency [100], in which *N*-ethyl-*N*-nitrosourea (ENU) random mutagenesis screens have yielded novel targets for therapy [101,102,103]. These examples were chosen because the SCA1 suppressor story can inform on experiments that show how SMN deficiency gives rise to SMA, whereas the Rett screen to suppress MECP2 deficiency can inform on possible additional targets for suppression of SMN deficiency.

SCA1 is caused by expansion of a CAG repeat in the *ataxin 1* gene (*ATXN1*), yielding a large glutamine repeat in the protein, which causes cerebellar and brain stem degeneration and ataxia [95]. Toxicity from the expansion repeat was proposed to occur through RNA aggregation, RAN translation, and/or disruption of various protein interactions [99]. Reducing the levels of the ATXN1 partner protein, cognate partner capicua (CIC) reduced severity in mouse models of SCA1 [97]. Furthermore, mutating *ATXN1* residues V591A and S602D, identified by structural studies followed by mutagenesis, resulted in abolishing the interaction with CIC in vitro and in vivo [98,99]. These mutations completely suppressed the SCA1 phenotype in mice, indicating that the toxicity caused by the expansion involves the critical partner CIC [99]. In essence, these studies eliminated the possibility that SCA1 toxicity resulted from RNA aggregation or RAN translation and demonstrate that toxicity is mediated by the aberrant interaction between mutant ATXN1 and CIC. CIC is a transcriptional repressor and alterations in the expression of some of its target genes, particularly ion channels, has been reported in SCA1 [104,105]. It remains unclear how these downstream targets contribute to the final phenotype of SCA1, however, those targets should be dependent on CIC repression. In a similar fashion, relevant downstream targets of SMN deficiency should directly link to SMN function. Moreover, like SCA1, similar screens in SMN partner proteins that interact with a mutant SMN can identify suppressors in SMA.

A second example is Rett Syndrome, which is caused by mutations that disrupt expression of the X chromosome encoded *MECP2* gene. Male wild-type C57/6J mice were mutagenized with ENU and crossed to female mice which have a null allele of *MECP2* (Mecp2^tm1.1Bird/+^). Male mice with the *Mecp2* null allele were then selected and assessed for correction. These rescued mice formed the founders of this dominant modifier screen, were bred for three generations, and assessed for the corrective phenotype. Modifiers were initially located using linkage analysis with quantitative trait mapping, followed by exome capture and sequencing. The exome alteration, an early stop codon, was found to overlap the *squalene epoxidase* (*SQLE*) gene, which is a rate-limiting enzyme in cholesterol synthesis [101]. This indicated that Rett mice had an abnormality in cholesterol metabolism; thus the authors tested if statins could modify the phenotype [101]. The statins are now in clinical trials for Rett; however, it should be noted that the alteration in cholesterol metabolism was not consistent in different strains of mice [106]. Thus, in humans there is the possibility that lipid metabolism could vary due to the individual’s genetic background [106,107].

Since the initial identification of *SQLE*, additional ENU screens have been performed using both mendelian models for quantitative linkage mapping and association analysis with linear regression [102]. Exome sequencing over a larger region subsequently revealed several groups of genes that work together to ameliorate phenotypic severity [102]. For instance, Rett mice heterozygous for both the early termination *SQLE* allele and an early termination mutant Rbb8 allele have a markedly improved lifespan of 203–813 days. While mutagenesis screens are often performed in *C. elegans*, Drosophila, and zebrafish, they are much less common in mice. Since the *Mecp2* gene is not found in invertebrates, these screens had to be performed in mice [101,106]. Screens such as this can also identify key elements and additional therapeutic targets in SMA. Unfortunately, thus far screens in *C. elegans* and Drosophila yielded suppressors that do not act in a similar fashion in SMA in mouse models. As in Rett Syndrome, association testing to identify multiple interacting loci is an excellent way to detect interacting loci as this technique considers the possibility of interacting mutants that may be informative in SMA. The SCA1 and Rett screens highlight how to identify suppressors or modifiers in neurodegenerative diseases with no clear target for effective therapy. In SMA we have a therapeutic target (SMN) thus our current quest is for additional therapeutic targets that act independently of SMN.

## 6. What Does SMN Do and What Does SMN Deficiency Affect

Reduction in SMN protein has been shown to affect many cellular and molecular pathways, including the biogenesis of the spliceosomal small nuclear ribonucleoproteins (snRNP) [108,109,110,111,112,113,114,115,116,117], U7 snRNP [118,119,120], telomerase [121,122,123], signal recognition particle (SRP) [124], translation regulation [125,126,127,128], and mRNA trafficking [129,130,131,132,133,134,135,136], of which there are many comprehensive reviews [137]. Unlike SMN, metabolic proteins like argininosuccinate lyase, glucocerebrosidase, iduronate-2-sulfatase, galactosylceramidase, and Wilson’s disease protein can be tested with biochemical assays that detect their enzymatic functions eliminating the need to rely on protein interactions to predict function. Multiple functions for SMN have been proposed due to protein interactions in immunoprecipitation experiments and protein alterations when SMN is deficient. The results of these experiments can be misleading if immunoprecipitations have low stringency or, in the case of alterations with SMN deficiency, if the effect is due to reduced SMN expression and not a downstream effector of SMN deficiency. For example, a measured effect of SMN deficiency could arise from altered splicing of a gene that then affects translation or mRNA trafficking. Proposed SMN functions can be broadly grouped into two classes: Sm-assembly dependent and Sm-assembly independent. We feel that identification of suppressors can help clarify the role SMN performs within these functions.

SMN is directly involved in the assembly of the heptameric Sm-ring onto the spliceosomal snRNAs and U7 snRNA, and likely involved in the premature Sm-assembled telomerase RNP prior to Lsm-ring assembly [108,109,110,112,117,118,119,120,122]. The most characterized of these assemblies is certainly snRNP biogenesis, in which SMN is associated with almost all steps from assembly of the Sm-ring onto snRNAs to delivery of the snRNP to the spliceosome [87,110,111,112,113,116,121,138,139,140,141,142,143,144,145,146,147,148,149,150,151,152,153,154,155,156,157]. There are functional, biochemical assays showing SMN direct involvement in snRNP assembly, both in vivo and in vitro [82,87,110,153,158,159]. Additionally, similar assays show SMN direct involvement in Sm-ring assembly onto U7 snRNA in vivo [120]. In the case of telomerase, SMN was not shown to directly assemble the Sm-ring onto telomerase RNA. However the anti-Sm immunoprecipitations capture premature telomerase RNA in *pombe* [122]. Interestingly, this complex was shown to be transient, in which the Sm-ring is removed and replaced by an Lsm-ring, making a mature and active telomerase. As SMN is the only protein known to assemble Sm-rings, to date, SMN is invoked to perform this assembly. In addition to functional assays for spliceosomal and U7 snRNP assembly, a specific SMN complex can be isolated from cells that is responsible for this biochemical activity [87,110,160]. In the case of functions that are not Sm-assembly dependent, there are currently no biochemical assays for the proposed function that are clearly directed by SMN, nor is there clear characterization of the complex that performs this function; thus, it is difficult to determine what role they play in SMA.

The canonical SMN complex that functions in snRNP assembly consists of SMN, Gemin 2–8, and Unrip [111,113,116,150]. Immunoprecipitations using anti-SMN or various anti-Gemin antibodies always pull down this complex, clearly indicating it is the predominant SMN complex in cells [108,110,148,160,161,162,163,164,165,166,167]. Indeed, with a Flag tag on Gemin2, a complex can be relatively easily purified which performs snRNP assembly [87,110,160]. This snRNP assembly activity is tightly correlated to the amount of SMN present [158,159]. Gemin2, 3, and 8 directly bind SMN, with Gemin2 binding in the N-terminus within exon 2A and Gemin8 in the C-terminus [88,115,160,168,169,170]. It is currently unknown what region of SMN is responsible for binding Gemin3. Importantly, SMN missense mutations show defects in assembling this complex as well as in the ability to assemble snRNPs. For example, Gemin8 binding was recently shown to require higher order SMN oligomer formation, which is affected by the SMNT274I mild missense mutation [88]. Furthermore, Gemin8 binding is entirely abolished in SMNH273R mutant homomers [88]. The SMN missense mutation D44V lies within the SMN-Gemin2 interface as constructed by NMR [115]. Though it does not abolish interaction with Gemin2, the D44V mutation is suggested to destabilize the integrity of the SMN complex as SMND44V homomers fractionate at lower molecular weight complexes than those made by wild-type SMN in vitro [87]. There are also mutant SMN proteins which do not affect complex formation but affect SMN complex function. Particularly, SMNE134K can form stable SMN complexes, but showed decreased ability to assembly snRNPs as compared to wild-type SMN in vitro [87]. The bulk of these experiments have been carried out in in vitro systems due to the added complexity of interrogating these questions in vivo. However, we have shown that the heteromeric oligomers of complementing SMN missense mutants are functionally equivalent at snRNP assembly as homomeric oligomers consisting of wild-type SMN in *Smn^−/D7^* iMEFs [77]. A caveat to this rule includes all SMNE13K heteromeric oligomers, which are less efficient at assembling snRNPs than wild-type SMN homomers [77]. This is interesting as it suggests SMNE134K loss of function is involved in snRNP assembly, parroting the in vitro experiments.

SMN is a ubiquitously expressed protein that clearly performs an essential function in assembling spliceosomal snRNPs. Why is it that SMN deficiency causes motor neuron death and not the death of other cell types in SMA? One theory is SMN has specific functions pertaining to motor neurons and this logic has led to trafficking proteins and mRNAs down motor neuron axons for further processing at the axon terminal [129,130,131,132,133,135,171,172,173]. Importantly, these functions are said to occur in an Sm-independent fashion [124,131,174,175,176]. The signal recognition particle (SRP) is a ribonucleoprotein complex that recognizes a specific N-terminal sequence of newly synthesized peptides, particularly for transmembrane proteins and stalls their translation until they are correctly docked with the endoplasmic reticulum [177,178,179]. Immunoprecipitations of native SMN complexes were shown to pull down the 7S RNA of the SRP [124]. This binding was shown to specifically involve Gemin5 as 7S RNA could still associate in conditions that abolished SMN complex association as well as with recombinant purified Gemin5 [124]. Furthermore, and more importantly, anti-Sm immunoprecipitations with the Sm-specific Y12 antibody did not yield 7S RNA, suggesting SMN complex association with SRP is independent of Sm-ring assembly [124]. It is suggested that the SMN complex completes the final assembly of SRP biogenesis by assembling SRP54 onto the premature 7S RNP complex, however mechanisms furthering this theory have yet to be discovered [124]. Additionally, SMN missense mutations have not been analyzed in this association, which may be useful in uncovering the role of SMN in SRP biogenesis.

Working in a similar pathway to SRP, SMN has been shown to associate with the alpha-COP subunit of the COPI coatomer complex, important for trafficking proteins from the *cis* end of the Golgi back to the rough endoplasmic reticulum for further packaging, processing, and trafficking in membrane-coated vesicles [180]. An SMN and alpha-COP interaction was first reported to occur in a yeast two-hybrid screen, then confirmed in immunoprecipitations in neuronal-like cells in culture [180,181]. Alpha-COP was shown to specifically interact with lysine residues in SMN exon 2B in vitro [181,182]. When these lysines are mutated to alanines, the SMN–alpha-COP interaction is abolished [182]. Mutations in alpha-COP were also shown to remove the SMN interaction [182]. It was shown that overexpression of alpha-COP was capable of rescuing growth of axons in cell culture and zebrafish, but only increased survival of SMA mice from 11 to 18 days with no motor functions assessed [182,183,184]. This interaction is interesting, as our experiments have shown that expression of an Smn lacking exon 2B, which contains the lysine residues responsible for alpha-COP interaction, completely rescues survival and snRNP assembly in *Smn^−/D7^* iMEFs [77]. These data suggest that the alpha-COP interaction is not essential to Smn function. As in the example for SCA1, where a mutant protein removed the ability to bind CIC resulted in removal of the toxic action of the glutamine expansion in ATXN1, we can ask if expression of an Smn which lacks the ability to bind alpha-COP modifies the motor outcomes and survival in SMA mice. It should be noted that an experiment regarding this question has been published, however expression from the mutated SMN transgene is quite low and this mutant has not been assayed for the ability to perform snRNP assembly [184]. Furthermore, if the *Smn* lacking exon 2B is crossed onto an *Smn* null, it can be determined whether the resulting mouse presents with an overt phenotype.

SMN has been reported in axons in RNPs that are not associated with Sm proteins [131,174] though time resolved quantitative proteomics have found SMN and SmB trafficking together in neuronal axons [185]. The composition of these RNPs has been largely uncharacterized. However one major interactor has been implicated: HuD (ELAV-like protein 4) [130,132,186]. HuD is a neuron-specific RNA-binding protein shown to increase the half-life of its AU-rich target mRNAs [132]. SMN–HuD interaction was first reported by expressing tagged SMN Tudor Domains in MN-1 cells, which pulled down HuD in an RNA-dependent manner [132]. HuD and SMN complex components were then shown to co-localize in the axons of neuronal cultures [132]. Overexpression of HuD resulted in the suppression of axonal growth defects in SMA zebrafish and neuronal cultures [132,186]. Interestingly, expression of the SMNE134K mutant in SMN deficient neuronal cultures and SMA zebrafish was shown to abolish HuD association [132,186]. This is a rather surprising feature of HuD–SMN binding as the SMNE134K appears to act in a dominant manner, in which the SMN heteromer should still be able to interact with HuD through the present wild-type SMN. This would suggest the suppression of the SMA phenotype observed in culture and zebrafish is working in an SMN-independent mechanism and is thus downstream of the critical function of SMN in regards to SMA. However, this can be addressed using suppressor screens, in which identifying a suppressor mutation in HuD that rescues SMNE134K–HuD interaction, or a specific mutation in SMN that disrupts HuD interaction, but not other SMN functions could clearly show SMN involvement in HuD-mediated mRNP trafficking.

Lastly, we discuss a possible role of SMN in regulation of translation. SMN has been shown to co-sediment with ribosomes, in what are called “SMN primed” ribosomes, and there is a decrease in fractional abundance of ribosomes in polysomes between control and SMA mice [127,128]. Particularly interesting is that there is a decrease in the relative abundance in ribosomal proteins in SMA animals as compared to wild-type [127]. However, SMN levels correlate with cell proliferation and the decrease in these proteins could be downstream of the critical function of SMN [187,188]. The SMN–ribosome interaction is further confounded by experiments in which recombinant SMN is incubated with an 80S ribosomal pellet [128]. Recombinant SMN is detected in relatively equivalent proportions in both unbound and bound fractions, regardless of whether SMN is incubated with the 80S ribosomal pellet, thus indicating a nonspecific association between SMN and ribosomes [128]. The SMN complex is stable under high salt conditions (500 mM NaCl) and sediments across 60S–80S fractions; therefore, an 80S ribosomal pellet likely contains the entire SMN complex and co-sedimentation is due to size, rather than protein binding events [160,175]. Lastly, an enrichment in mRNAs containing 5′UTRs with IRES motifs were shown to be preferentially affected by SMN deficiency [128]. Interestingly, Gemin5 is known to bind and negatively regulate translation of mRNAs with IRES motifs in their 5′UTRs, and also to directly interact with ribosomal subunits L3, L4, eIF3B, eIF4B, and eIF4E [176,189,190,191,192,193]. Thus, the direct role of SMN in translation is largely uncharacterized and speculative. However, we see immense value in these experiments, as the authors show intriguing changes in response to SMN deficiency with a clear mechanism to how those changes arise. We look forward to further characterization of these SMN–ribosome complexes and suggest that identification of SMN mutants that abolish ribosomal association while retaining SMN complex formation would be informative.

**Figure 3:**
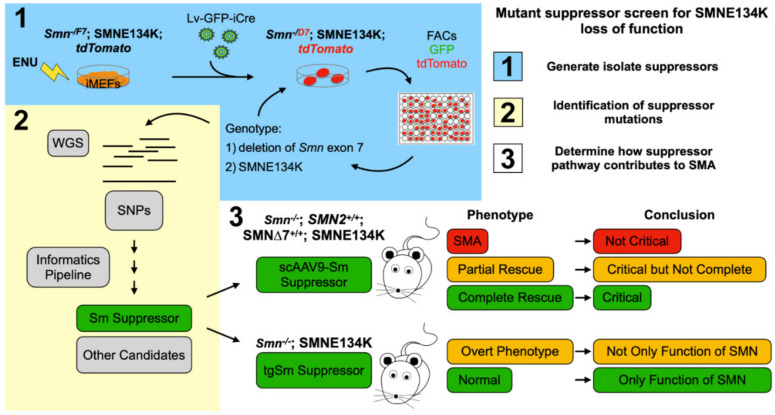
Mutant suppressor screen for SMNE134K loss of function. (**1**) Pictorial representation of the random mutagenesis screen and isolation of SMNE134K mutant suppressor lines. *Smn^−/F7^*;SMNE134K iMEFs were mutagenized with *N*-ethyl-*N*-nitrosourea (ENU) then treated with a lentivirus delivering iCre-recombinase and Green Fluorescent Protein (GFP) to delete *Smn* exon 7 from the *Smn^F7^* allele (now *Smn^D7^*). iCre activity is monitored by the removal of a floxed-stop codon upstream of tdTomato, activating tdTomato expression. Cells were sorted by FACs to enrich the tdTomato and GFP double positive population of cells and were seeded at low density. Surviving colonies were picked, expanded, and genotyped for retention of SMNE134K and deletion of *Smn* exon 7 using ddPCR. Cell populations which showed greater than 70% deletion of *Smn* exon 7 were kept and pushed to 100% deletion by additional lentivrial transfection of iCre and dilution cloning. (**2**) Whole genome sequencing (WGS) was performed on surviving *Smn^−/D7^*;SMNE134K mutant suppressor lines to identify variant SNPs not common to the unmutagenized *Smn^−/F7^*;SMNE134K cell lines. Resultant variant SNPs were prioritized in a candidate list and culminated in testing whether a mutation in an Sm protein was responsible for suppression of *Smn^−/D7^*;SMNE134K lethality. This Sm Suppressor indicates Sm-assembly is the essential function of SMN in iMEFs. (**3**) Outline of experiments that should determine whether rescue of Sm-assembly is critical to SMA. In ∆7 SMNE134K SMA mice, an AAV9 delivering the Sm suppressor can result in three outcomes: no modification of the SMA phenotype, a partial rescue, or complete rescue. Additionally, transgenic expression of the Sm suppressor in an *Smn* null, SMNE134K-expressing mouse can have two outcomes: development of an overt phenotype or normalcy. Each of these outcomes suggests a different conclusion, thus showing how suppressor screens can inform on the critical function of SMN regarding SMA.

It is possible to identify suppressors of specific SMN missense mutations that inform which function of SMN is critical to SMA. In this regard, we have used the SMNE134K and performed a random mutagenesis screen, using our *Smn^−/F7^* iMEFs to ask whether we can make a mutation that rescues SMNE134K loss of function. This experiment is pictorially represented in Figure 3. In this experiment, *Smn^−/F7^*;SMNE134K iMEFs are mutagenized with ENU, then full-length wild-type Smn is removed by transfection with a lentivirus delivering an improved Cre-recombinase (iCre), yielding *Smn^−/D7^*;SMNE134K. Thus, there is no functional Smn from the mouse locus and the cells are entirely reliant on the nonfunctional SMNE134K, which causes the cells to die [77]. However, if a mutation in a gene occurs that can restore the essential function lost by SMNE134K, the cells should live. Indeed, we have performed such a screen and we have identified several mutant suppressor lines which we have sequenced to locate the suppressor. Importantly, the rescue is dependent on having SMNE134K present. If suppression of *Smn^−/D7^*;SMNE134K lethality occurs in a specific function of SMN, i.e., Sm-assembly, the suppressor, and SMNE134K can then be introduced into SMA mice or Smn null mice to ask the question, what rescue occurs? Indeed, we know that suppressors in Sm proteins can be obtained. Do Smn null mice expressing SMNE134K, and the suppressor show complete rescue of motor neurons, all phenotypes, or do they still show phenotypic abnormality? If it is complete rescue, then Sm-assembly is the critical function of SMN. If partial rescue occurs, then Sm-assembly contributes to SMA, but other functions must also contribute. Lastly, if a true SMA phenotype results, then there is a new mouse model of SMA and Sm-assembly is not the critical pathway in SMA. Though we have used the SMNE134K missense mutant, this same suppressor screen can be extended to other SMN missense mutations as none rescue survival of the *Smn^−/D7^* iMEFs. These experiments can inform on the specific functional interaction of other members of the SMN complex, other unique SMN complexes, and how they work in an unbiased manner.

## 7. Downstream Targets of SMN

The above section identifies the function of SMN that is critical for SMA. Once these are defined, it becomes possible to look for specific therapeutic targets or downstream targets of SMN deficiency. Currently we would say that targets are ill-defined and unclear in all cases. An example is the recently reported B-Raf which is implied to be critically impaired and result in motor neuron death [194]. The primary evidence given is that the neurotrophic pathway that supports motor neurons is reduced in presymptomatic mice. The first question that arises is how specific this is to SMA and whether this also occurs in SMA with respiratory distress (SMARD) or some other motor neuron disease control. This is required to know whether this neurotrophic pathway is specific to SMA or has the chance of being a secondary change. In addition, B-Raf null mice appear to have loss of both sensory neurons and motor neurons, begging the question as to whether the null really models the situation in SMA [195]. In this instance, identifying a hypomorphic allele would help clarify the situation as to whether B-Raf is required at higher levels in motor neurons. In addition, while sensory defects have been reported in SMA mice, it is not clear there is death of sensory neurons in mice or SMA patients. Lastly, the importance of the B-Raf pathway was confirmed using the *C. elegans* model of SMA and has yet to be replicated in mice or iPS cells [194]. We stress the importance of translating these experiments into mammalian models. Additionally, the authors state this will give access to a SMN-independent therapy strategy, but the reason for B-Raf reduction, if related to SMA, is deficiency of SMN. The report also states that the B-Raf change has not been directly related to SMN deficiency and could very well be downstream of the primary defect. This is problematic and we feel an approach that first defines the important pathway and then analyzes that pathway is essential to a logical path forward.

Given SMN role in spliceosomal snRNP biogenesis, it is plausible that a reduction in snRNP abundance could lead to inefficient or aberrant pre-mRNA splicing [196]. Many in the SMA community adhere to a stance that we have tested the gamut for splicing changes and since nothing has moved forward this cannot be the critical function of SMN regarding SMA. We feel the data on altered splicing is simply not adequate. Firstly, the most homogenous sample of mature motor neurons that has been collected for splicing analysis was performed on two SMA and two normal mice, with large variations in the levels of exon incorporation. Surely two disease, two controls, and no neurodegenerative disease control, with large variation between samples cannot be considered adequate. Additional studies have been performed in iPS motor neurons; however, these are not mature motor neurons, and it is not clear these cells are an accurate substitute [197,198]. However, before moving forward with sequencing experiments looking for splicing changes, the critical function of SMN needs to be defined and then the downstream target, like altered splicing, can be looked for. In addition, the downstream target should be significantly modified by SMN reduction and correcting this downstream target should restore a specific aspect of the SMA phenotype. In the context of an additive therapy to SMN restoration, the downstream targets should not be considered as an additive therapy of SMN without evidence that this is the case. Most will be SMN dependent and restored by SMN. Therefore, much work is needed in identifying downstream targets of SMN deficiency.

## 8. Modifiers of the SMA Phenotype

In SMA the phenotype is largely determined by the copy number of *SMN2*, but there are exceptions that diverge from this rule. The first is discussed above, regarding mild missense mutations in *SMN1*. Second, there are variants that lie within the *SMN2* gene that alter the incorporation of SMN exon 7 and result in the production of more full-length SMN from that particular *SMN2* gene [22,199,200,201,202]. Lastly, there are exception cases that would appear to not involve either *SMN1* or *SMN2*, and thus the variant must be located outside the SMA region. Since mild missense mutations in *SMN1* are discussed above, we will focus on modifiers within *SMN2* as well as modifiers outside the SMA locus in this section.

A strong example of modifiers in *SMN2* is the c.859G > C variant located in exon 7 [200,201]. This variant increases the inclusion of exon 7 in the *SMN2* transcripts and has never been reported in a Type 1 individual with two copies of *SMN2*. The c.859G>C variant is found in Type 2 or 3 patients [199,200,201]. The variant has also been reported in a Type 2 individual who has two copies of *SMN2* and is heterozygous for the variant. Whereas in other cases which also have two copies of the variant is in a homozygous state, SMA Type 3b results [199]. *SMN2* variants in intron 6 that alter the incorporation of exon 7 are also associated with a milder than expected phenotype [22,202]. Thus, at least one mechanism of modification is altering the amount of full-length SMN coming from *SMN2*. However, there are discordant siblings in which the *SMN2* gene(s) are identical between the siblings [22,32,203,204,205,206,207]. In our experience, these discordant siblings are most common in three copy *SMN2* cases and predominantly conform to a Type 2 for the severe sibling and a Type 3b, or milder, in the other sibling [22]. However, there are exceptions that occur in all SMA types [208] and thus a model that can be used for testing in the general population is defined as Type 1 with two copies of *SMN2*, Type 2 with three copies, and Type 3 with four copies which can be used to help identify these modifiers. We suggest that the modifiers that occur in discordant siblings are the same as in the general population such that with a sufficiently large population or when using an isolated population with a particular founder, association studies can be used to identify the critical areas where these modifiers lie.

The first modifier proposed for SMA that lies outside the SMA region was *plastin 3* (*PLS3*). *PLS3* was found to be more highly expressed in lymphoblasts isolated from mild or unaffected siblings in discordant families. *PLS3* is located on the X chromosome, and thus was reported as a sex-dependent, partially penetrant modifier [207]. However, the partially penetrant phenotype without description of the *PLS3* mutation responsible for this increase in expression is troubling. Furthermore, SMA families in which the more severe sibling has higher *PLS3* expression than the mild sibling have been reported [209]. Consequently, some have suggested that modifiers only function in milder SMA cases, however, there are siblings with Type 1 and 2 SMA, as well as cases where the male sibling is less severely affected than their female sibling [12,208].

The overexpression of *PLS3* has been studied in two severe SMA mouse models, Taiwanese SMA mice and ∆7SMA mice, with opposing results [210,211]. Transgenic overexpression in our hands in the ∆7SMA model did not alter the SMA phenotype including the electrophysiological properties of the NMJ [211]. A previous publication suggests modification by *PLS3* only occurs in mild SMA, in which a low dose of ASO directed against ISS-N1 was administered to the Taiwanese SMA mice to elevate SMN levels and create a milder phenotype [207]. However, the results from these experiments are hard to interpret as the overexpression of *PLS3* itself can alter the endocytosis pathways and alter uptake of the ASO [210,212,213,214]. Additionally, the *PLS3* mutation, rs871773, that increases *PLS3* expression in colon cancer does not associate with mild SMA exception cases [22,215]. Until there is more definitive data, we favor a more cautious approach that increased *PLS3* expression is not responsible for the modification of the phenotype.

The second reported modifier of SMA, Neurocalcin Delta (*NCALD*), was found in a single family from a genome-wide linkage analysis. This analysis identified eight genomic regions that could possibly link with the milder SMA case, however none of these regions reached the required significance level to determine linkage [216]. Subsequent expression analysis revealed reduced expression of *NCALD* [216]. However, it is unclear if one of the other seven regions is also involved in impacting phenotypic severity. One mutation, a 17 base-pair deletion adjacent to a punitive super enhancer, was found 600 kilobases upstream of the *NCALD* gene, however this deletion on its own does not modify the SMA phenotype as it is clearly found in both mild and severe discordant siblings [22]. A second mutation, a CT insertion in intron 1 of the *NCALD* gene, [216] is predicted to be a strong cryptic splice site that might inhibit *NCALD* expression [22]. Again though, this variant also occurs in the severe sibling of a discordant pair and in a concordant sibling pair [22]. While it is possible both variants must occur on the same allele to modify the phenotype, it seems more likely that the predicted cryptic splice site causes reduced *NCALD* expression and the 17 bp deletion adjacent to the enhancer is merely a rare polymorphic variant. [216] As *NCALD* mutations occur in both mild and severe siblings more experiments are required to determine if *NCALD* really modifies the SMA phenotype in humans.

There are several other genes that have been reported to modify the SMA phenotype in mice, *C. elegans*, and *Drosophila*, but in no case has the candidate been shown to modify the phenotype in humans by an association analysis [217,218,219,220,221]. While expression analysis of potential modifying genes is important, establishing a direct genetic link to the phenotypic alteration is paramount in identifying true modifiers. Ideally, a large pool of well characterized genomic SMA patient samples should be used to test for candidate association or for analysis between concordant and discordant siblings. As a rule, the modifying variant should only associate with the mild SMA case and not in the severe or concordant siblings. These variants can be further tested in a large panel of patient samples (not siblings) that are divided according to their *SMN2* copy. The model of *SMN2* copy number that we have used states that two copies of *SMN2* predicts Type 1 SMA, three copies of *SMN2* predicts Type 2 SMA, and four copies of SMN2 predicts Type 3 SMA. Any sample not conforming to this copy number model was considered to be discordant. One can then perform an association analysis to test if variant show a statistically significant association with a milder than expected phenotype. This would indicate that the variant is a true phenotypic modifier [22].

Alternatively, studying a population derived from a limited number of founder individuals, such as the Mennonite, Amish, and Hutterite SMA patients, can be a highly effective way to identify phenotypic modifiers. For example, a common haplotype marking a *SMN1* deletion with two copies of *SMN2* on one chromosome occurs in the Hutterites. Some individuals homozygous for this haplotype have a deletion of *SMN1* with four copies of *SMN2* and display no obvious SMA phenotype while other have typical Type 3 SMA [222]. Comparison of these two groups of individuals with whole genome sequencing will likely reveal an additional chromosomal region containing modifier genes.

## 9. Conclusions

The first era of genetic discovery in SMA identified the causative *SMN1* gene and the therapeutic *SMN2* target that led to three incredibly effective FDA approved drugs in a remarkably short period of time. The future of genetic studies, in conjunction with biochemistry, will demonstrate how SMN missense mutations can disrupt the function of SMN through complementation. Studying the SMNA2G and SMNT274I mutations that do not complement will likely identify new interaction between the N- and C-terminus of SMN. Finally, the use of partially functional SMN mutants will identify suppressors of specific SMN missense mutations in novel protein binding partners. Identification of true modifiers of the SMA phenotype will provide additional targets for therapy to improve outcomes of symptomatic SMA patients. Together genetic and biochemical studies have the potential unravel the basic biology of the disease and give us a clearer understanding of the function of SMN that is critical to the development of SMA.

## Figures and Tables

**Table 1:** Loss of function missense mutations in *SMN1* identified in SMA patients.

Mutation	SMN Exon	SMN Domain	SMA Type	*SMN2* Copy Number	Reference
A2G	1		3	1	Parsons *Am. J. Hum. Genet*. 1998 [62]
A2V	1		3	1	Vinette *Poster Abstract* 2008 [63]
G26D	1		2–3	1	Vinette *Poster Abstract* 2008 [63]
D30N	2A	Gemin2	2	2	Sun *Hum. Mutat*. 2005 [64]
D44V	2A	Gemin2	3	1	Sun *Hum. Mutat*. 2005 [64]
W92S	3	Tudor	1	3	Kotani *J. Neurol*. 2007 [65]
V94G	3	Tudor	2	3	Clermont *Hum. Mutat*. 2004 [66]
G95R	3	Tudor	3	1	Sun *Hum. Mutat*. 2005 [64]
G106S	3	Tudor	1	2	Vinette *Poster Abstract* 2008 [63]
A111G	3	Tudor	1–2	2	Sun *Hum. Mutat*. 2005 [64]
I116F	3	Tudor	1	2	Cusco *Neurology* 2004 [67]
Y130C	3	Tudor	3	2	Mihal *Poster Abstract* 2007 [68]
E134K	3	Tudor	1	2	Sun *Hum. Mutat*. 2005 [64], Clermont *Hum*. *Mutat*. 2004 [66]
Q136E	3	Tudor	1	1	Cusco *Neurology* 2004 [67]
D140V	3	Tudor	2–3	1–2	From personal communication with Thomas Prior [69]
A188S	4		1	ND	Zapletalova *Neuromuscul. Disord*. 2007 [70]
P245L	6	Oligomerization	3	ND	Rochette *Neurogenetics* 1997 [71]
L260S	6	Oligomerization	2	2	Clermont *Hum. Mutat*. 2004 [66]
S262G	6	Oligomerization	3	1	Sun *Hum. Mutat*. 2005 [64]
S262I	6	Oligomerization	3	1	Parsons *Am. J. Hum. Genet*. 1998 [62], Hahnen *Hum. Mol. Genet*. 1997 [72]
M263R	6	Oligomerization	1	2	Clermont *Hum. Mutat*. 2004 [66]
M263T	6	Oligomerization	2	1	Alias *Hum. Genet*. 2009 [73]
S266P	6	Oligomerization	2	2	Mihal *Poster Abstract* 2007 [68]
Y272C	6	Oligomerization	1	2	Hahnen *Hum. Mol. Genet*. 1997 [72]
H273R	6	Oligomerization	2	ND	Mihal *Poster Abstract* 2007 [68]
T274I	6	Oligomerization	3	1	Hahnen *Hum. Mol. Genet*. 1997 [72]
G275S	6	Oligomerization	3	ND	Clermont *Hum. Mutat*. 2004 [66]
G275D	6	Oligomerization	1	1	Vinette *Poster Abstract* 2008 [63]
G279C	7		1	ND	Wang *Neurogenetics* 1998 [74]
G279V	7		1	ND	Talbot *Hum. Mol. Genet*. 1997 [75]
Q282A	7		NA	NA	Carrel *J. Neurosci*. 2006 [76]
E286A	7		NA	NA	Carrel *J. Neurosci*. 2006 [76]

NA is not applicable, as Q282A and E286A are synthetic alleles and were not identified in the SMA patient population. ND is not determined, as in the *SMN2* copy number was not determined in the patients described and references in table correspond to these indices [62,63,64,65,66,67,68,69,70,71,72,73,74,75,76].

## Data Availability

Not applicable.
